# The Contamination of Commercial ^15^N_2_ Gas Stocks with ^15^N–Labeled Nitrate and Ammonium and Consequences for Nitrogen Fixation Measurements

**DOI:** 10.1371/journal.pone.0110335

**Published:** 2014-10-17

**Authors:** Richard Dabundo, Moritz F. Lehmann, Lija Treibergs, Craig R. Tobias, Mark A. Altabet, Pia H. Moisander, Julie Granger

**Affiliations:** 1 University of Connecticut Avery Point, Department of Marine Sciences, Groton, CT, United States of America; 2 University of Basel, Institute for Environmental Geosciences, Basel, Switzerland; 3 University of Massachusetts Dartmouth, Department of Estuarine and Ocean Sciences, North Dartmouth, MA, United States of America; 4 University of Massachusetts Dartmouth, Department of Biology, North Dartmouth, MA, United States of America; Reader in Inorganic Chemistry, United Kingdom

## Abstract

We report on the contamination of commercial 15-nitrogen (^15^N) N_2_ gas stocks with ^15^N-enriched ammonium, nitrate and/or nitrite, and nitrous oxide. ^15^N_2_ gas is used to estimate N_2_ fixation rates from incubations of environmental samples by monitoring the incorporation of isotopically labeled ^15^N_2_ into organic matter. However, the microbial assimilation of bioavailable ^15^N-labeled N_2_ gas contaminants, nitrate, nitrite, and ammonium, is liable to lead to the inflation or false detection of N_2_ fixation rates. ^15^N_2_ gas procured from three major suppliers was analyzed for the presence of these ^15^N-contaminants. Substantial concentrations of ^15^N-contaminants were detected in four Sigma-Aldrich ^15^N_2_ lecture bottles from two discrete batch syntheses. Per mole of ^15^N_2_ gas, 34 to 1900 µmoles of ^15^N-ammonium, 1.8 to 420 µmoles of ^15^N-nitrate/nitrite, and ≥21 µmoles of ^15^N-nitrous oxide were detected. One ^15^N_2_ lecture bottle from Campro Scientific contained ≥11 µmoles of ^15^N-nitrous oxide per mole of ^15^N_2_ gas, and no detected ^15^N-nitrate/nitrite at the given experimental ^15^N_2_ tracer dilutions. Two Cambridge Isotopes lecture bottles from discrete batch syntheses contained ≥0.81 µmoles^ 15^N-nitrous oxide per mole ^15^N_2_, and trace concentrations of ^15^N-ammonium and^ 15^N-nitrate/nitrite. ^15^N_2_ gas equilibrated cultures of the green algae *Dunaliella tertiolecta* confirmed that the ^15^N-contaminants are assimilable. A finite-differencing model parameterized using oceanic field conditions typical of N_2_ fixation assays suggests that the degree of detected ^15^N-ammonium contamination could yield inferred N_2_ fixation rates ranging from undetectable, <0.01 nmoles N L^−1 ^d^−1^, to 530 nmoles N L^−1 ^d^−1^, contingent on experimental conditions. These rates are comparable to, or greater than, N_2_ fixation rates commonly detected in field assays. These results indicate that past reports of N_2_ fixation should be interpreted with caution, and demonstrate that the purity of commercial ^15^N_2_ gas must be ensured prior to use in future N_2_ fixation rate determinations.

## Introduction

Nitrogen (N) is a major nutrient required universally by photosynthetic organisms. Its availability in the environment can directly affect the ecology and productivity of terrestrial and marine ecosystems, with important implications for the regional and global carbon cycles. The natural input of bioavailable N to the biosphere is dominated by nitrogen fixation, the biological reduction of dinitrogen (N_2_) gas to ammonium (NH_4_^+^). Two methods are commonly utilized to measure N_2_ fixation rates in the field, the ^15^N_2_ tracer assay [1] and the acetylene (C_2_H_2_) reduction assay [2,3]. The ^15^N_2_ tracer assay was originally developed when artificially ^15^N-enriched substrate N_2_ first became available [4]. This approach was then superseded by the acetylene reduction technique, as the cost and availability of high precision isotope ratio measurements proved restrictive [3]. The acetylene reduction assay, however, is associated with variations in the factor used to convert C_2_H_2_ reduction into N_2_ equivalents, and with potentially biasing effects of C_2_H_2_ on the physiology of N_2_ fixing organisms, among other issues [5]–[7]. Interest in the ^15^N_2_ tracer assay later regained momentum, owing to the increased affordability of Isotope Ratio Mass Spectrometry (IRMS) instrumentation and to concurrent developments in ^15^N tracer techniques. Today, it is generally the preferred method to quantify N_2_ fixation rates in both terrestrial and aquatic environments [1], owing to its high sensitivity, and ability to provide qualitative and quantitative constraints on the translocation and the fate of biologically fixed N [8]–[10].

A salient strength of the ^15^N_2_ tracer assay is that ^15^N-enrichment detected in biomass can be ascribed to the biological reduction of N_2_ exclusively, as no interfering processes can carry out the reduction of ^15^N_2_ gas concurrently. This premise requires that the ^15^N_2_ stock be devoid of any contaminant ^15^N-species that could be assimilated into biomass simultaneously. However, during recent research projects on N_2_ fixation conducted independently in our laboratories at the University of Connecticut Avery Point and the University of Massachusetts Dartmouth, convergent observations indicated that some commercial ^15^N_2_ stocks could be contaminated with ^15^N-enriched N-species other than N_2_, including nitrate, nitrite and/or ammonium. These reactive forms of N would be readily assimilated by microorganisms, leading to significantly biased (i.e., overestimated) N_2_ fixation rate measurements.These observations motivated the current study, with the goal of testing whether commercially available ^15^N_2_ stocks contain ^15^N-contaminants at levels that would interfere with ^15^N_2_ tracer N_2_ fixation assays, particularly in the open ocean, and to assess if such contaminants are prevalent among ^15^N_2_ stocks from different suppliers. We thus uncovered substantial contamination of one of three brands of commercial ^15^N_2_ gas with bioavailable inorganic ^15^N-species. Our findings raise important concerns regarding the pervasiveness of reactive ^15^N contamination of the ^15^N_2_ stocks, and the extent to which these contaminants may have affected the magnitude of the N_2_ fixation rate estimates reported in the literature. We outline steps to contend with this issue to ensure the veracity of future N_2_ fixation estimates.

## Methods

### Reagents

Four 33 mL lecture bottles of 98+ at% ^15^N-labeled N_2_ gas were purchased from Sigma-Aldrich (produced by their subsidiary, Isotec Stable Isotopes; St. Louis, MO; Stock Keeping Unit 364584), three from lot # SZ1670V, synthesized in 2010, and one from lot # MBBB0968V, synthesized in 2014. Two 1L lecture bottles of 98+ at% ^15^N_2_ were purchased from Cambridge Isotopes (Tewksbury, MA, part # NLM-363-1-LB) from respective lot #’s I1-11785A and I-16727. One 1L lecture bottle of 98+ at% ^15^N_2_ was purchased from Campro Scientific (Berlin, Germany; catalogue # CS01-185_261) from lot # EB1169V. Ammonium and nitrate solutions were prepared with salts or with solutions obtained from different distributors: sodium nitrate (NaNO_3_: CAS 7631-99-4), potassium nitrate (KNO_3_: CAS 7757-79-1), and ammonium chloride (NH_4_Cl: CAS 12125-02-9) from Fisher Scientific (Pittsburgh, PA); analytical-grade potassium nitrate (CAS 7757-79-1) from Fluka Analytical and a gravimetric solution of ammonium chloride (catalogue # AS-NH3N9-2Y) from SPEX CertiPrep (Metuchen, NJ).

### Preparation of nitrate and ammonium solutions equilibrated with ^15^N_2_ gas

In order to determine whether the ^15^N_2_ gas stocks contained ^15^N-labeled ammonia (NH_3_) or nitrate and/or nitrite (NO_x_) contaminants, aqueous solutions of natural abundance (unlabeled) ammonium and nitrate salts were equilibrated overnight with an air headspace supplemented with an injection of ^15^N_2_ gas. After equilibration, the ^15^N/^14^N ratio of ammonium and the ^15^N/^14^N and ^18^O/^16^O ratios of nitrate/nitrite in solution were measured, as well as the ^15^N/^14^N ratio of N_2_ gas in the headspace, as described below. The isotope ratios of nitrate and ammonium were compared to those in control solutions, which were not supplemented with ^15^N_2_ gas. Experiments with the Campro Scientific ^15^N_2_ stock were verified for ^15^N-nitrate/nitrite contaminants only (and not for ^15^N-ammonium).

Initial experiments consisted of 40 mL or 100 mL solutions of 10, 50, 100, 200, or 300 µmol L^−1^ nitrate and 5 µmol L^−1^ ammonium chloride in 60 mL or 120 mL serum vials that were sealed with Thermo Scientific gas-impermeant stoppers (part # C4020-30) or with Bellco Glass septum stoppers (catalogue # 2048-11800). The 20 mL of air headspace in each of the treatment vials was supplemented with 0.1 mL of ^15^N_2_ gas from respective bottles from each of the three suppliers (three lecture bottles from Sigma-Aldrich lot # SZ1670V and one bottle from lot # MBBB0968V, two bottles from Cambridge Isotopes lot # I1-11785A and lot # I-16727, and one bottle from Campro Scientific lot # EB1169V). The solutions were equilibrated overnight on a shaker, after which the ^15^N/^14^N and ^18^O/^16^O isotope ratios of nitrate were analyzed as described below. The ^15^N/^14^N isotope ratio of ammonium was also analyzed (described below) in experimental solutions treated with the Sigma-Aldrich and Cambridge Isotopes stocks, but not the Campro Scientific stock.

The experimental sensitivity to ^15^N-contaminants was increased in subsequent experiments involving ^15^N_2_ stocks that did not show clear evidence of contamination in the experiments described above (see *Results*) by increasing the volume of ^15^N_2_ gas injections and decreasing solution volumes. Experiments were initiated in which 2 mL ^15^N_2_ gas was equilibrated overnight in 20 mL serum vials containing 10 mL solutions of 10 µmol L^−1^ sodium nitrate, after which the ^15^N/^14^N and ^18^O/^16^O ratios of nitrate were measured as described below. Similarly, 10 mL solutions of 5 µmol L^−1^ ammonium chloride were dispensed in 20 mL serum vials and equilibrated overnight with 2 mL ^15^N_2_ gas, after which the ^15^N/^14^N isotope ratios of ammonium were analyzed (described below).

The measured ^18^O/^16^O ratios of nitrate/nitrite in solutions equilibrated with ^15^N_2_ gas from some stocks suggested the presence of ^46^N_2_O contamination. As our analyte for isotope ratio analysis is N_2_O, and ^46^N_2_O can be explained by both ^15^N^15^N^16^O and ^14^N^14^N^18^O, N_2_O that is doubly labeled with ^15^N is falsely detected as δ^18^O_NO3_ enrichment. The presence of ^46^N_2_O contamination in^ 15^N_2_ gas was verified directly for one of the Sigma-Aldrich stocks (Lot # SZ1670V) by adding 0.0125, 0.020, or 0.025 mL of ^15^N_2_ stock to 20 mL serum vials containing 10 nmoles of reference N_2_O in helium. The N and O isotopic composition of the N_2_O was analyzed as described below, and compared to unamended N_2_O injections.

### Dunaliella tertiolecta cultures

The marine green alga *Dunaliella tertiolecta* was cultured in growth media equilibrated with ^15^N_2_ gas in order to ascertain the susceptibility of ^15^N-labeled gas contaminants to assimilation by non-N_2_-fixing organisms. Culture medium was prepared from filtered Long Island Sound sea water supplemented with 50 µmol L^−1^ NaNO_3_, 36.3 µmol L^−1^ NaH_2_PO_4_*H_2_O, and 107 µmol L^−1^ Na_2_SiO_3_*9H_2_O, as well as f/2 trace metals and f/2 vitamins [11], added from filter sterilized stock solutions. Medium (200 mL) was dispensed in 250 mL stoppered glass bottles. Experimental treatment bottles were equilibrated overnight with 0.2 mL ^15^N_2_ gas from either a Cambridge Isotopes (lot #I-16727) or Sigma-Aldrich (lot # SZ1670V) lecture bottle. Following inoculation, cultures were left loosely capped and placed on a windowsill with exposure to natural light. Nitrate concentrations were monitored daily. Upon the complete depletion of nitrate, 8 days after inoculation, the cultures were harvested on pre-combusted GF/F filters. Filters were dried at 60°C for 18 h pending N isotopic analysis of the particulate nitrogen (described below).

### Nitrate and ammonium concentrations

Nitrate concentrations in the experimental solutions were verified *via* reduction to nitric oxide in hot vanadium (III) solution followed by detection with a chemiluminescence NO_x_ analyzer (model T200 Teledyne Advanced Pollution Instrumentation) [12]. Ammonium concentrations were measured by derivatization with orthophthaldialdehyde (OPA) and fluorometric detection on an AJN Scientific f-2500 Fluorescence Spectrophotometer [13].

### Nitrate N and O isotope ratio analyses

Nitrate/nitrite nitrogen (^15^N/^14^N) and oxygen (^18^O/^16^O) isotope ratios were measured using the denitrifier method [14,15]. Nitrate (and nitrite) in experimental samples was converted stoichiometrically to nitrous oxide (N_2_O) by a denitrifying bacterial strain (*Pseudomonas chlororaphis* f. sp. *aureofaciens*, ATCC 13985) that lacks nitrous oxide reductase. The N and O isotopic composition of N_2_O was then measured on a Delta V Advantage Isotope Ratio Mass Spectrometer (IRMS) interfaced with a modified Gas Bench II gas chromatograph (Thermo Fisher) purge and trap system. The isotope ratio measurements are reported in the conventional delta (δ) notation in per mille (‰) units, defined for N and O by the following equations:
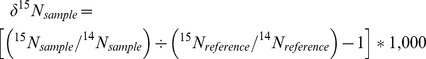


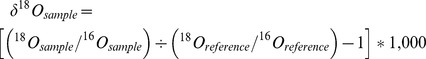



The ^15^N/^14^N reference is N_2_ in air, and the ^18^O/^16^O reference is Vienna Standard Mean Ocean water (V-SMOW). Individual analyses on the GC-IRMS were referenced to injections of N_2_O from a pure N_2_O gas cylinder, and then standardized through comparison to the international nitrate standards USGS-34 (δ^15^N of −1.8‰ *vs.* air; δ^18^O of −27.9‰ *vs.* V-SMOW), USGS-32 (δ^ 15^N of +180‰ *vs.* air; δ^18^O of +25.7‰ *vs.* V-SMOW), and IAEA-NO-3 (δ^15^N of +4.7‰ *vs.* air; δ^18^O of +25.6‰ *vs.* V-SMOW) [16]–[18], using standard bracketing techniques. Nitrate samples from experiments with Campro Scientific ^15^N_2_ were standardized with USGS-32 and IAEA-NO-3, and an additional internal lab nitrate standard (UBN-1; δ^15^N of 14.15‰ *vs.* air; δ^18^O of +25.7‰ *vs.* V-SMOW). Precision for analytical replicates was ≤0.2‰ for δ^15^N_NO3_ and ≤0.2‰ for δ^18^O_NO3_ for isotope ratio amplitudes encompassed by the standards. Above 200‰, the precision decreased in proportion to δ^15^N_NO3_ amplitude, with standard deviations of 7.5‰ for δ^15^N_NO3_ at or above 1000‰. Similarly, precision decreased with increasing δ^18^O_NO3_, with standard deviations of 1.3‰ to 6.1‰ for δ^18^O_NO3_ values ≥80‰. The poor precision of the higher range measurements is likely due to the variable contribution of a trace NO_X_ contaminant in denitrifier preparations with δ^15^N and δ^18^O values that are in the range of natural abundance samples [19].

### Nitrous oxide N and O isotope ratio analyses

N_2_O isotope ratios were measured directly on the GC-IRMS, and referenced against the N_2_O tank, which was standardized indirectly by comparison to the δ^15^N and δ^18^O of nitrate standards.

### Ammonium N isotope ratio analyses

The ammonium δ^15^N_NH4_ was measured using the hypobromite-azide method [20]. Ammonium in basic solution was converted to N_2_O *via* oxidation to nitrite (NO_2_^−^) with hypobromite, followed by reduction of nitrite to N_2_O with sodium azide in acetic acid. The δ^15^N of the N_2_O analyte was measured on the GC-IRMS, as outlined above. Measurements were calibrated using solutions made from the international standard ammonium salts, IAEA-N1 and IAEA-N2, with assigned δ^15^N values of +0.4‰, +20.3‰ *vs.* air, respectively [16,17,21,22]. Our standard error for analytical replicates was ≤0.6‰ at relatively low ^15^N-abundances, but increased substantially for δ^15^N_NH4_ from 100‰ to 9000‰, varying from 2.9‰ to as high as 59.7‰. As with the nitrate analyses, the low precision of higher range measurements likely stems from the variable contribution of a trace ammonium or nitrite contaminant with a natural abundance δ^15^N value, inadvertently introduced during the analyses.

### Headspace N_2_ isotope ratio analyses

To measure the δ^15^N of N_2_ gas in the headspace of experimental samples, 75 µL of headspace was injected into 12 mL Exetainer vials previously flushed with helium, then analyzed on a Gas Bench II GC-IRMS (Delta V Advantage *Plus*) operated in continuous flow mode. N_2_ and (O_2_+ Ar) were separated on a 5-Å mole-sieve capillary gas chromatography column. The analyses were standardized with parallel analyses of ambient N_2_ gas in air. These direct N_2_ gas measurements were carried out for experiments conducted using two of three lecture bottles from Sigma-Aldrich lot # SZ1670V, and for experiments conducted using the lecture bottle from Cambridge Isotopes lot # I1–11785A. The ^15^N_2_ concentration in the headspace of other experiments was estimated from the tracer injection volume rather than from direct measurements.

### Particulate nitrogen isotope ratio analyses

The δ^15^N of particulate nitrogen (PN) was analyzed using a Costech Instruments elemental combustion system (model 4010) coupled to a Thermo Scientific Delta V Advantage IRMS. Analyses were standardized using L-glutamic acid reference materials, USGS-40 (δ^15^N of −4.52‰ *vs.* air) and USGS-41 (δ^15^N of +47.57‰ *vs.* air) [23].

## Results

**Figure 1:**
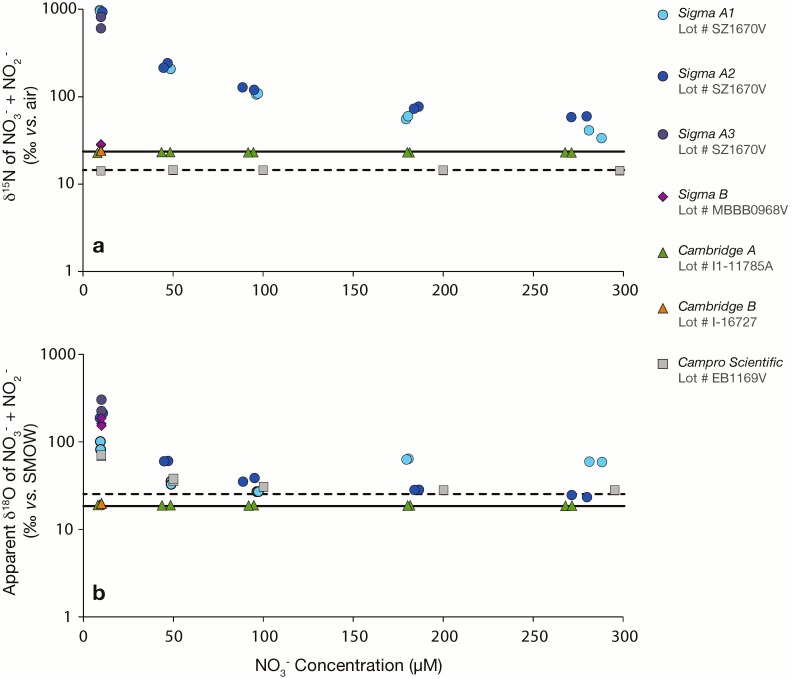
(**a**) δ^15^N_NO3+NO2_ (log scale) of nitrate solutions (10–300 µmol L^−1^) following equilibration with 0.1 mL ^15^N_2_ gas from lecture bottles procured from three distributors. Solutions were 40 mL for Sigma-Aldrich and Campro Scientific equilibrations, and 100 mL for Cambridge Isotopes equilibrations. The solid line corresponds to the δ^15^N_NO3_ of the control solutions for Sigma-Aldrich and Cambridge Isotopes experiments (δ^15^N_NO3_ = 23.5±0.5‰); the dashed line corresponds to controls for Campro Scientific experiments (δ^15^N_NO3_ = 14.15±0.1‰). Paired symbols identify replicate experimental treatments. (**b**) Corresponding apparent δ^18^O_NO3+NO2_ of the experimental nitrate solutions. The solid line corresponds to the δ^18^O_NO3_ of control solutions for the Sigma-Aldrich and Cambridge Isotope experiments (δ^18^O_NO3_ = 18.9±0.3‰); the dashed line corresponds to controls for Campro Scientific experiments (25.4±0.3‰).

**Figure 2:**
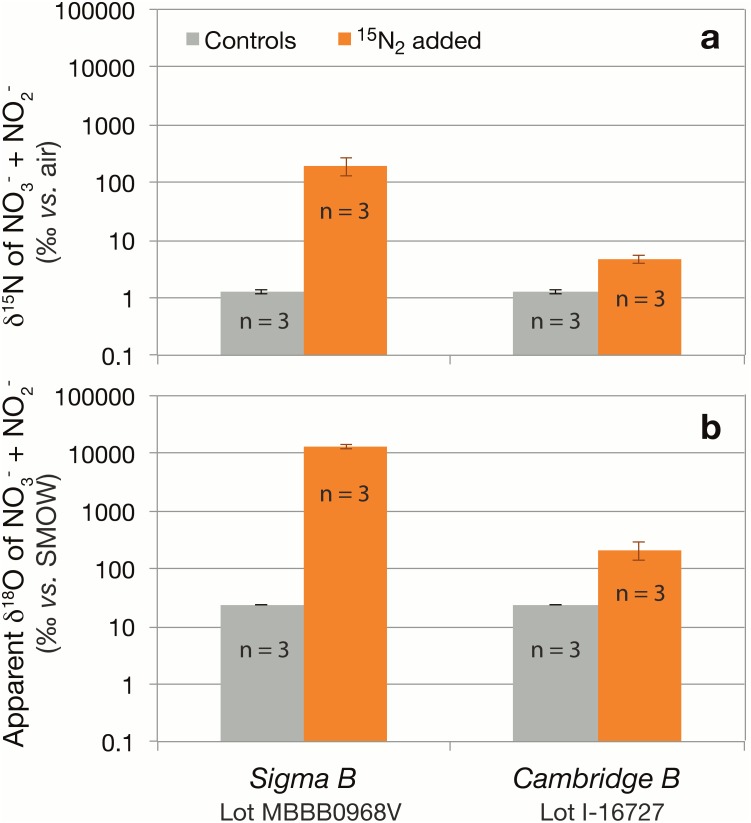
(a) δ^15^N_NO3+NO2_ (log scale) of higher sensitivity equilibrations of 10 µmol L^−1^ nitrate solutions (10 mL) with 2 mL of ^15^N_2_ gas from a Cambridge Isotopes or a Sigma-Aldrich bottle. (**b**) Corresponding apparent δ^18^O_NO3_ (log scale) of higher sensitivity equilibrations of the two stocks. n = the number of experimental replicates.

Nitrate solutions equilibrated with any of three ^15^N_2_ gas stocks from Sigma-Aldrich lot # SZ1670V (referred to hereafter as *‘Sigma A1, A2 and A3’*) showed a substantial increase in the δ^15^N of nitrate (and possibly nitrite) compared to control solutions in the lower sensitivity nitrate dilutions (Fig. 1a). Respective ^15^N enrichments evidenced by the δ^15^N_NO3+NO2_ were inversely proportional to the concentration of nitrate in the solutions, from nearly 1000‰ at 10 µmol L^−1^ nitrate to 30‰ at 290 µmol L^−1^ nitrate, compared to a δ^15^N_NO3_ of 23.5±0.5‰ in the corresponding potassium nitrate control solutions. The ^15^N enrichments imparted on the nitrate solutions were comparable among the three lecture bottles from this lot (# SZ1670V). The δ^15^N_NO3+NO2_ resulting from equilibration with a single Sigma-Aldrich gas stock from lot # MBBB0968V (*‘Sigma B’*) was relatively modest, but still significant, averaging 28.4±0.3‰ at 10 µmol L^−1^ nitrate, compared to 23.5±0.5‰ in the corresponding control solutions (Fig. 1a). When tested at the more sensitive experimental dilution, equilibrations of nitrate solutions with the *Sigma B* stock resulted in a δ^15^N_NO3+NO2_ of 200.2±70.9‰, compared to a δ^15^N_NO3_ of 1.3±0.1‰ in control solutions (Fig. 2a). These measurements thus indicate that N_2_ gas stocks sourced from lot # SZ1670V contained 410±80 µmoles of ^15^N-nitrate and/or nitrite per mole of ^15^N_2_, whereas the bottle from lot # MBBB0968V contributed 1.8±0.6 µmoles of ^15^N-nitrate and/or nitrite per mole of ^15^N_2_ (Table 1). The ^15^N-nitrate additions were determined by a mass balance calculation:
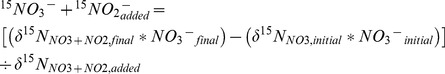
where ^15^NO_3_^−^ + NO_2_^−^_added_ is the moles of ^15^N-labeled nitrate and/or nitrite added by the ^15^N_2_ gas injection, δ^15^N_NO3+NO2,added_ is presumed to be equivalent to the δ^15^N of ^15^N_2_ tracer gas (266,540‰), NO_3_^−^_initial_ and NO_3_^−^_final_ refer to the moles of nitrate in solution before and after ^15^N_2_ equilibration, and δ^15^N_NO3_,_initial_ and δ^15^N_NO3+NO2_,_final_ refer to the δ^15^N_NO3_ of nitrate solutions before and after ^15^N_2_ equilibration. The quantity of ^15^N_2_ gas added to experimental treatments was measured explicitly in *Sigma A1* and *A2* bottles, and was calculated from the ^15^N_2_ injection volumes for experiments treated with *Sigma A3* and *B* stocks.

**Table 1:** The quantity of ^15^N-labeled contaminants detected relative to ^15^N_2_ additions.

µmoles ^15^N_X_ per mole ^15^N_2_			
	^15^NO_3_^−/^NO_2_^−^	^15^NH_4_^+^	^46^N_2_O
*Sigma A1*lot # SZ1670V	420±110	34±11	≥21±3
*Sigma A2*lot # SZ1670V	420±40	520±30	109±5§
*Sigma A3* †lot # SZ1670V	350±80	N/A	≥63±15
*Sigma B* †lot # MBBB0968V	1.8±0.6	1900±560	≥49±17
*Cambridge A*lot # I1-11785A	n.d.*	0.052±0.020	n.d.*
*Cambridge B* †lot # I-16727	0.024±0.006	0.014±0.004	≥0.81±0.24
Campro Scientific†lot # EB1169V	n.d.*	N/A	≥11±3

The µmoles of ^15^N contaminants (NO_3_^−^+NO_2_^−^, NH_4_^+^, and N_2_O) detected per mole of ^15^N_2_ gas from lecture bottles provided by different suppliers. N/A = not available; n.d. = not detected.

*Not explicitly tested in high sensitivity ^15^N_2_ dilutions.

† Moles of ^15^N_2_ estimated from the injection volume rather than direct measurements.

§ ^46^N2O measured directly.

In contrast to the Sigma-Aldrich stocks, ^15^N_NO3+NO2_ contaminants were significantly lower, or possibly absent, in Cambridge Isotopes and Campro Scientific ^15^N_2_ stocks. The δ^15^N_NO3+NO2_ values of solutions treated with Cambridge Isotopes ^15^N_2_ gas (lots # I1–11785A and I-16727, hereafter referred to as *Cambridge A* and *Cambridge B*, respectively) and with Campro Scientific ^15^N_2_ gas (lot # EB1169V) were indistinguishable from those of control solutions at all experimental nitrate concentrations in the lower sensitivity tests (Fig. 1a). In the more sensitive experimental treatments, however, solutions treated with *Cambridge B* ^15^N_2_ gas (lot # I-16727) had a δ^15^N_NO3+NO2_ of 4.8±0.8‰, compared to a δ^15^N_NO3_ of 1.3±0.1‰ in control solutions (Fig. 2a). This stock thus contributed trace contaminants on the order of 0.024±0.006 µmoles of ^15^N-nitrate and/or nitrite per mole of ^15^N_2_ (Table 1). Nitrate isotope ratios in the *Cambridge A* (lot # I1–11785A) and Campro Scientific ^15^N_2_ gas stocks were not tested at these lower experimental dilutions.

In treatments using several ^15^N_2_ gas stocks, δ^18^O_NO3_ was found to be elevated relative to control solutions. The denitrifier method, employed for δ^18^O_NO3_ measurements, involves the bacterial reduction of NO_3_^−^ and NO_2_^−^ to N_2_O, and the subsequent analysis of N_2_O using an IRMS. However, the elevated δ^18^O_NO3_ values detected within experimental treatments are expressly *not* explained by the formation of ^14^N^14^N^18^O during bacterial reduction of ^15^N-enriched nitrate, which could only account for a negligible portion of the observed δ^18^O_NO3+NO2_ increase. Instead, the values are best explained by the presence of doubly-labeled ^15^N^–^N_2_O (i.e., ^46^N_2_O) in the ^15^N_2_ gas stocks. The apparent δ^18^O_NO3+NO2_ of nitrate solutions equilibrated with all of the Sigma-Aldrich stocks, the Campro Scientific stock, and the *Cambridge B* ^15^N_2_ stock proved to be greater than that of control solutions in the low sensitivity treatments (Fig. 1b). At 10 µmol L^−1^ nitrate, the apparent δ^18^O_NO3+NO2_ of treated solutions was 188.5±83.8 among the *Sigma A1-A3* stocks (lot # SZ1670V), 169.8±17.9‰ for the *Sigma B* stock (lot # MBBB0968V), and 20.1±0.2 for the *Cambridge B* stock, compared to 18.9±0.3‰ in corresponding control solutions. The apparent δ^18^O_NO3+NO2_ of the Campro Scientific stock at 10 µmol L^−1^ nitrate was 70.4±1.4‰, compared to 25.7±0.1‰ in corresponding control solutions. The apparent δ^18^O_NO3+NO2_ of the samples decreased coherently with increasing nitrate concentrations for respective stocks. The apparent δ^18^O_NO3+NO2_ values of solutions equilibrated with the *Cambridge A* stock, at 19.1±0.2‰, were not distinguishable from the control solutions. In the more sensitive equilibrations, nitrate solutions equilibrated with *Sigma B* ^15^N_2_ gas had a δ^18^O_NO3+NO2_ of 13,129±1186‰ compared to 23.9±0.2‰ in control solutions, whereas the δ^18^O_NO3+NO2_ of the *Cambridge B* stock was 216.7±78.4‰ (Fig. 2b). Given that the apparent δ^18^O_NO3_ enrichments are explained by the presence of ^46^N_2_O, the inverse relationship between δ^18^O values and nitrate concentration stems from the fact that the detected ^46^N_2_O derives from ^46^N_2_O dissolved in the nitrate solutions, and solutions containing higher nitrate concentrations require lower sample volume injections when using the denitrifier method for IRMS analysis. The observed excess ^46^N_2_O levels indicate ^15^N^15^N^16^O contaminants (µmole ^46^N_2_O per mole of ^15^N_2_) on the order of 41±21 among the *Sigma A1-A3* bottles, 49±17 in *Sigma B*, 11±3 in Campro Scientific, and 0.81±0.24 in *Cambridge B* (Table 1). The *Cambridge A* bottle was not tested in higher sensitivity dilutions that could have revealed the presence of some N_2_O therein. The presence of ^46^N_2_O contaminant was verified directly for the *Sigma A2* lecture bottle from analyses of N_2_O amended with injections of ^15^N_2_ gas. Among four experimental samples, 109±5 µmoles of ^46^N_2_O were detected per mole of *Sigma A2* ^15^N_2_ added, more than double the ^46^N_2_O that was detected in samples analyzed by the denitrifier method (39±8 µmole ^46^N_2_O per mole of ^15^N_2_). This discrepancy likely resulted because samples analyzed by the denitrifier method were uncapped immediately prior to their injection into *P. aureofaciens* denitrifier cultures, allowing N_2_O to escape to the atmosphere. As contaminant N_2_O was not the target analyte of the denitrifier measurements, precautions were not taken to prevent N_2_O gas loss at this step. The ^46^N_2_O concentrations derived from solution equilibrations of respective ^15^N_2_ stocks thus constitute lower limits (Table 1).

**Figure 3:**
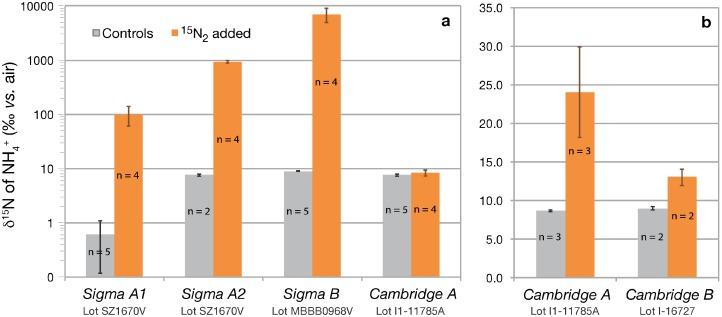
(a) δ^15^N_NH4_ (log scale) of 5 µmol L^−1^ ammonium solutions after equilibration with 0.1 mL ^15^N_2_ gas from respective Sigma-Aldrich and Cambridge Isotopes lecture bottles *vs.* control solutions. Sigma-Aldrich treatments utilized 40 mL ammonium solutions, whereas Cambridge Isotopes treatments utilized 100 mL ammonium solutions. (**b**) δ^15^N_NH4_ of higher sensitivity equilibrations of 5 µmol L^−1^ ammonium solutions (10 mL) with 2.0 mL ^15^N_2_ gas from Cambridge Isotopes lecture bottles *vs.* control solutions. n = the number of experimental replicates.

Solutions equilibrated with Sigma-Aldrich ^15^N_2_ gas showed substantial ^15^N-enrichments of ammonium compared to control solutions (Fig. 3a): Equilibration with ^15^N_2_ from the *Sigma A1* lecture bottle (lot # SZ1670V) resulted in a δ^15^N_NH4_ of 99±39‰, compared to 0.6±0.5‰ for the control solution (NH_4_Cl, SPEX CertiPrep); equilibration with ^15^N_2_ from the *Sigma A2* bottle (lot # SZ1670V) yielded a δ^15^N_NH4_ of 940±60‰, compared to 7.6±0.3‰ for the control solution (NH_4_Cl salt, Fisher Scientific); equilibration with ^15^N_2_ from the *Sigma B* bottle (lot # MBBB0968V) resulted in a δ^15^N_NH4_ of 7030±2100‰, compared to 9.0±0.06‰ for the corresponding control solutions (NH_4_Cl salt, Fisher Scientific). Mass balance calculations based on these isotope ratio values thus evidence the presence of 34±11, 518±26, and 1890±560 µmoles of ^15^N-ammonium per mole of ^15^N_2_ injected from *Sigma A1*, *A2*, and *B* ^15^N_2_ bottles, respectively (Table 1). Unlike ^15^N-labelled nitrate/nitrite contaminants, the ^15^N-ammonium contaminants appeared to be variable among bottles of lot # SZ1670V (Fig. 3a). The *Sigma A3* lecture bottle was not tested for ^15^N-ammonium.

In contrast to Sigma-Aldrich stocks, ammonium solutions equilibrated with ^15^N_2_ from the *Cambridge A* bottle had a δ^15^N_NH4_ of 8.3±1.0‰, comparable to that of the corresponding control solution of 7.6±0.3‰ (NH_4_Cl salt, Fisher Scientific) in the lower sensitivity experiments (Fig. 3a). In the more sensitive dilutions, however, ^15^N-ammonium contaminants were detected in both of the *Cambridge A* and *B* stocks (Fig. 3b). Solutions equilibrated with *Cambridge A* had a δ^15^N_NH4_ of 24.0±5.9‰, compared to 8.7±0.1‰ for the control solutions (NH_4_Cl salt, Fisher Scientific) and solutions equilibrated with *Cambridge B* had a δ^15^N_NH4_ of 13.1±1.1‰, compared to 9.0±0.1‰ for the control solutions ((NH_4_Cl salt, Fisher Scientific). The enrichment relative to control solutions invariably originates from a ^15^N-ammonium contaminant, and cannot be attributed to a trace N_2_O (^15^N^14^N^16^O) contaminant, because the samples were purged when conducting ^15^N-ammonium analyses, following the oxidation of ammonium to nitrite with hypobromite. These more sensitive treatments thus reveal the presence of minuscule ^15^N-ammonium concentrations in the Cambridge Isotopes stocks, on the order 0.052±0.020 and 0.014±0.004 µmoles of ^15^N-ammonium per mole of ^15^N_2_ gas in lots # I1–11785A and I-16727, respectively (Table 1).

The control solutions in the ^15^N-ammonium experiments prepared from a single Fisher Scientific NH_4_Cl salt stock revealed progressively heavier mean δ^15^N_NH4_ values among experiments, at 7.6±0.3‰, 8.7±0.1‰, or 9.0±0.06‰. The solutions with a δ^15^N_NH4_ estimated at 7.6±0.3‰ and 8.7±0.1‰ were made fresh from the salts for each experiment, such that the ^15^N-enrichment of ammonium cannot be attributed to the progressive degassing of ammonia in solution during storage. In turn, isotopic standards for NH_4_^+^ (IAEA-N1 and N2) were stored in acidic solution, and thus were not subject to progressive degassing. Moreover, degassing of the isotopic standards would manifest as progressively lower δ^15^N values measured for control solutions. Inter-batch variability intrinsic to the hypobromite-azide method [20] is plausible, as this technique is relatively recent, such that subtle sensitivities may not yet be apparent.

**Figure 4:**
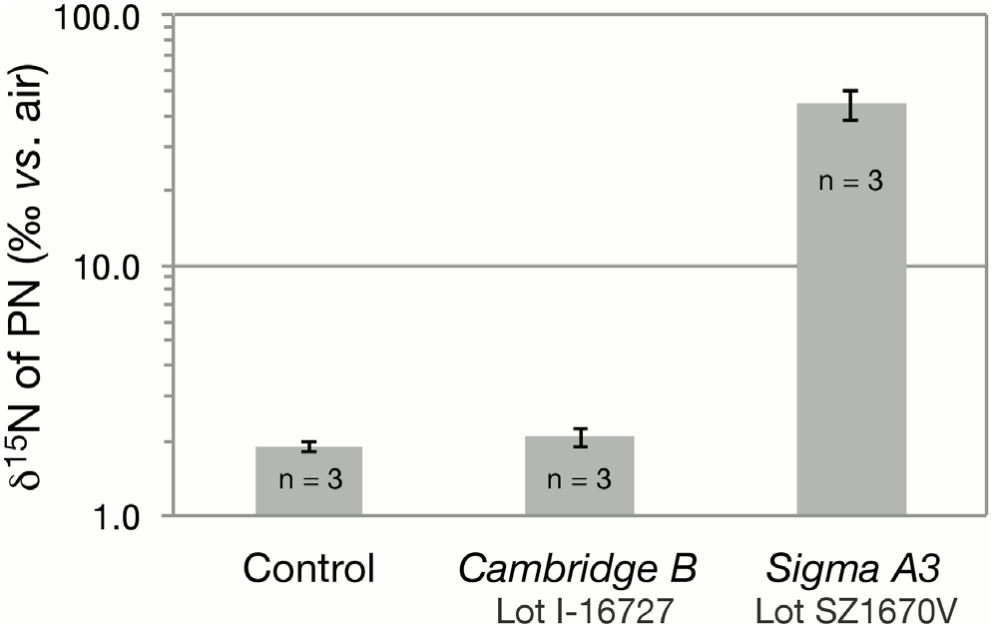
The δ^15^N of particulate nitrogen (δ^15^N_PN_) of *D. tertiolecta* harvested in stationary phase following growth in media containing sodium nitrate (and no ammonium) and equilibrated with ^15^N_2_ gas from Sigma Aldrich or Cambridge Isotopes. n = the number of experimental replicates.

*D. tertiolecta* cultures grown in medium equilibrated with ^15^N_2_ gas from the *Sigma A3* bottle expectedly showed substantial ^15^N enrichment of particulate nitrogen (δ^15^N_PN_), averaging 44.3±6.1‰ among triplicate treatment cultures compared to 1.9±0.1‰ in control cultures (Fig. 4). Conversely, the δ^15^N_PN_ of cultures equilibrated with *Cambridge B* ^15^N_2_ gas was 2.1±0.2‰, and thus not detectably different from that of control cultures, at 1.9±0.1‰ (Fig. 4).

## Discussion

This study reveals that some commercial ^15^N_2_ gas stocks contain contaminant ^15^N-labeled bioavailable nitrogen species, including nitrate/nitrite, ammonium and nitrous oxide. Substantial levels of ^15^N-labeled nitrate/nitrite, ammonium, and nitrous oxide were detected in Sigma-Aldrich stocks from lot # SZ1670V. Another Sigma-Aldrich stock from a different lot (# MBBB0968V) contained considerably less, but still significant, ^15^N-nitrate/nitrite contaminants, similar nitrous oxide concentrations, and a greater concentration of ^15^N-ammonium. Cambridge Isotopes stocks, in turn, contained relatively low concentrations of ^15^N-nitrate/nitrite, ^15^N-ammonium and ^15^N-nitrous oxide. A ^15^N_2_ stock from Campro Scientific contained no detected ^15^N-nitrate/nitrite contaminants in low sensitivity experiments, but measurable ^15^N-nitrous oxide. Indeed, a certificate of analysis provided by Campro Scientific attests that stocks may contain up to 15 ppm N_2_O. ^15^N-ammonium was not analyzed in Campro Scientific gas, nor was the stock tested at more sensitive ^15^N_2_ dilutions, which could have revealed trace ^15^N-nitrate or ammonium contaminants in the stock. In any case, ^15^N-contamination with nitrous oxide is of no obvious consequence for biological ^15^N_2_ applications, such as N_2_ fixation rate measurements. However, the presence of ^15^N-nitrate, nitrite and ammonium has serious implications for measurements of N_2_ fixation, as these contaminants could lead to the detection of false positives or inflated rates.

The propensity of the detected ^15^N-labeled contaminants to be assimilated into biomass was verified directly from cultures of *D. tertiolecta,* which acquired elevated δ^15^N of PN in media equilibrated with a Sigma-Aldrich ^15^N_2_ stock. Expectedly, media equilibrated with ^15^N_2_ from a Cambridge Isotopes stock did not cause detectable ^15^N-enrichment of biomass. At the given experimental conditions, however, the complete assimilation of the contaminant ^15^N-nitrate/nitrite from the Sigma-Aldrich stock should have yielded greater δ^15^N_PN_ values than those observed, of at least 56.3±5.4‰ (*vs.* control δ^15^N_PN_ values of ∼1.9‰), notwithstanding the additional contribution of any ^15^N-ammonium contaminant (^15^N-ammonium was not measured explicitly in the *Sigma A3* stock). This discrepancy is difficult to reconcile. We tentatively posit that ^15^N-*nitrite* comprises a substantial fraction of the trace ^15^N-nitrate/nitrite contaminant, and that *D. tertiolecta* may not be able to transport nitrite at nanomolar to sub-nanomolar concentrations. Indeed, such trace nitrite concentrations are likely below the thresholds achievable by micro-algal nitrite transport systems [24].

The contaminants in the ^15^N_2_ stocks ostensibly derive from the method of ^15^N_2_ gas production. ^15^N_2_ gas is generally produced by the catalytic oxidation of ^15^N ammonia (^15^NH_3_) gas with cupric oxide [25]. The bulk of the oxidation product is N_2_ gas, although more oxidized N species are also produced in lesser quantities, specifically N_2_O and NO [26]. Thus, potential contaminants in a ^15^N_2_ gas stock would expectedly consist of unreacted ammonia gas, N_2_O, and nitric oxide (NO). In contact with any oxygen and water vapor, NO would inadvertently be oxidized to nitric and nitrous acid [27], which would, in turn, dissociate to nitrate and nitrite upon dissolution in water, respectively. Purification of the ^15^N_2_ gas from unreacted ammonia and from the generated nitrogen oxides involves sequential acid and alkaline scrubbing, respectively [28] and/or cryo-trapping of ammonia and NOx gases. Upon personal communication, Cambridge Isotopes and Campro Scientific did not provide details on their method of ^15^N_2_ production, whereas Sigma-Aldrich reported that the company’s subsidiary, Isotec, produces ^15^N_2_ gas by the catalytic oxidation of ^15^N-ammonia gas with cupric oxide, followed by sequential rounds of cryo-trapping and alkaline scrubbing of the N_2_ gas to increase purity.

In order to gauge the extent to which the observed ^15^N-ammonium contamination of Sigma-Aldrich and Cambridge Isotopes ^15^N_2_ gas could skew estimates of N_2_ fixation in incubations with ^15^N_2_ gas, we modeled a field incubation experiment in which microorganisms assimilate the ^15^N-ammonium contaminant rather than reduce ^15^N_2_ gas. A simple finite-differencing model of a ‘typical’ oceanic N_2_ fixation assay was devised, in which 0.1 mL of ^15^N_2_ gas was equilibrated in two different water sample volumes, 0.25 L or 4.5 L, then incubated for 24 hours. Prescribed biomass and growth rates were characteristic of those at the oligotrophic surface ocean, namely, a particulate N stock of 0.2 µmol L^−1^ assimilating ammonium at a specific growth rate coefficient (µ) of 0.1 d^−1^ to 0.3 d^−1^, with a recycling rate (the rate at which particulate N is returned to the ammonium pool) equivalent to the respective growth rate. The prescribed δ^15^N of the initial particulate N was 0‰ [29], and the δ^15^N of ambient ammonium was −2‰ [30]. Incremental initial concentrations of ambient ammonium were prescribed, from 1 nmol L^−1^ to 1 µmol L^−1^. Ammonium concentrations in surface oligotrophic waters are typically very low (≤10 nmol L^−1^), however, ammonium is a pervasive contaminant that could easily be introduced during sample preparation, as well as leached from incubation vial septa. We note that the ^15^N-ammonium introduced by the ^15^N_2_ gas, while substantial in terms of the ^15^N/^14^N ratio of ammonium, is on the order of ∼20 nanomolar *at most* (under the modeled conditions), and thus has minimal effect on ambient ammonium concentrations. ^14^N-ammonium contamination is expected to be negligible, given the method of ^15^N_2_ gas synthesis. Finally, ^15^N-ammonium assimilation was simulated for the broad range of ^15^N-ammonium contaminant concentrations observed among Sigma-Aldrich and Cambridge Isotopes lecture bottles. N_2_ fixation rates inferred from the simulated δ^15^N increase of particulate N were computed based on the formulation of Montoya (1996):
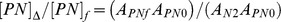


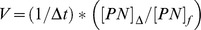


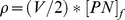
[PN]_Δ_ is the change in particulate nitrogen concentration, [PN]_f_ is the final particulate nitrogen concentration, *A*_PN*f*_ is the final ^15^N enrichment of particulate nitrogen, *A*_PN0_ is the initial ^15^N enrichment of particulate nitrogen, *A*_N2_ is the ^15^N enrichment of the N_2_ available for fixation, V is the specific rate of N_2_ uptake, and ρ is the volumetric rate of N_2_ fixation.

The model-derived ‘N_2_ fixation rates’ resulting from Sigma-Aldrich ^15^N-ammonium contaminant levels ranged from undetectable, <0.01 nmol N L^−1 ^d^−1^, to as high as 530 nmol N L^−1 ^d^−1^ under the modeled conditions (Table 2). Rates were clearly sensitive to the concentration of ^15^N-contaminant, the ambient ammonium concentration, the incubation volume, and the specific growth rate. At the lower level of ^15^N-ammonium contaminant observed in the Sigma-Aldrich stocks, N_2_ fixation rates were comparable to rates observed *in situ* for nearly all parameter permutations, from<0.01 to 9 nmoles N L^−1 ^d^−1^. N_2_ fixation rates reported for marine environments cover a broad range, from 0.01 nmoles L^−1 ^d^−1^ to tens of nmoles N L^−1 ^d^−1^ [31]–[33]. Rates simulated with the highest observed level of contaminant, in smaller incubation volumes at given ^15^N_2_ additions, and/or with low ambient ammonium concentrations, tended to surpass rates observed *in situ* by 10 to 100 fold. The N_2_ fixation rates modeled using the minute contaminant level detected in a Cambridge Isotopes stock ranged from undetectable to 0.02 nmoles N L^−1 ^d^−1^ (Table 2), approximating the lower limit of some N_2_ fixation rates reported in the literature [31],[34]–[38]. These simulated rates can be deemed conservative since the model does not account for any assimilation of contaminant ^15^N-nitrate/nitrite, and the 0.1 mL ^15^N_2_ injection volume used in the model is on the lower end of ^15^N_2_ injection volumes typically used in open ocean N_2_ fixation rate measurements.

**Table 2:** Inferred N_2_ fixation rates (nmoles N L^−1 ^d^−1^) resulting from ^15^N-labeled contaminants.

**Ambient [NH_4_^+^]**	**Cambridge Isotopes**			**Sigma-Aldrich**			**Sigma-Aldrich**			
**(µmol L^−1^)**	***Cambridge A***			***Sigma A1***			***Sigma B***			
	**Lot # I1–11785A**			**Lot # SZ1670V**			**Lot # MBBB0968V**			
	0.52 µmol ^15^NH_4_^+^/mol ^15^N_2_			25 µmol ^15^NH_4_^+^/mol ^15^N_2_			1900 µmol ^15^NH_4_^+^/mol ^15^N_2_			
	**µ = 0.1**	**µ = 0.2**	**µ = 0.3**	**µ = 0.1**	**µ = 0.2**	**µ = 0.3**	**µ = 0.1**	**µ = 0.2**	**µ = 0.3**	**Incubation Volume (L)**
**0.001**	0.019	0.019	0.019	9.0	9.0	9.0	310	460	530	**0.25**
**0.01**	0.014	0.016	0.016	7.5	8.5	8.6	250	400	470	
**0.1**	n.d.	n.d.	n.d	1.6	2.7	3.6	90	170	220	
**1**	n.d.	n.d.	n.d.	0.17	0.32	0.45	13	24	34	
**0.001**	n.d.	n.d.	n.d.	0.50	0.50	0.50	38	38	38	**4.5**
**0.01**	n.d.	n.d.	n.d.	0.42	0.47	0.48	30	35	36	
**0.1**	n.d.	n.d.	n.d.	0.08	0.15	0.19	6.5	11	15	
**1**	n.d.	n.d.	n.d.	n.d.	0.012	0.017	0.7	1.4	1.9	

N_2_ fixation rates that would be inferred from 24-h field N_2_ fixation assays conducted with ^15^N_2_ stocks containing the respective concentrations ^15^N-ammonium contaminants detected in Sigma-Aldrich and Cambridge Isotopes ^15^N_2_ gas. In the simulations, microbial plankton assimilate ^15^N-ammonium rather than fix ^15^N_2_. Incubations are simulated in volumes of 0.25 L or 4.5 L equilibrated with 0.1 mL of ^15^N_2_ gas, with 2.0×10^−7^ µmol L^−1^ of plankton nitrogen (with a δ^15^N = 0‰) assimilating at a range of specific growth rates, µ (d^−1^), countered by equivalent recycling rates, at incremental concentrations of ambient ammonium (δ^15^N_NH4_ = −2‰). Inferred rates of <0.01 nmoles N L^−1 ^d^−1^ are considered undetectable (n.d.).

Based on the simulations above, the likelihood of N_2_ fixation rates being inflated when using contaminated ^15^N_2_ gas stocks is high. It is surprising, then, that contamination of the ^15^N_2_ stocks has not been reported previously. While growth solely upon N from N_2_ fixation would eliminate the effect of ^15^N-labeled bioavailable contaminants, it is expected that nitrate and ammonium assimilation would be rapid relative to N_2_ fixation due to the prohibitive energetic cost of N_2_ fixation [39]. A review of pertinent literature reveals that soil scientists were once aware of the possible contamination of ^15^N_2_ with bioavailable N, and took steps to mitigate it [28,40]. However, to the best of our knowledge, there is no mention of potential contamination of ^15^N_2_ stocks in the marine literature, or in more recent terrestrial literature. The fact that this issue has gone unnoticed could mean that major contamination of ^15^N_2_ gas stocks, such as that observed here in Sigma-Aldrich stocks, could be limited to the current lots. Supporting the notion that contamination is rare is the observation of undetectable N_2_ fixation rates at the surface ocean, where phytoplankton readily assimilate ammonium [38] – even in investigations utilizing the Sigma-Aldrich (Isotec) ^15^N_2_ gas [31]. However, a representative at Isotec stated that their procedures for synthesis and purification of ^15^N_2_ gas have not changed in past decades, which suggests that ^15^N-contaminants may have been pervasive in previous lots. Failure to detect interferences from ^15^N-contaminants in previous studies may then stem from incubation conditions conspiring to yield expected rates of *apparent* N_2_ fixation in spite of the presence of ^15^N contaminants (Table 2). Interference of ^15^N contaminants on N_2_ fixation rate measurements may then be relatively minor in systems where bioavailable N assimilation rates are low and/or where ambient nitrate and ammonium concentrations are relatively elevated (≥100 nmol L^−1^; Table 2), as ambient assimilable N effectively diminishes ^15^N enrichment resulting from ^15^N-labeled contaminants.

It is difficult, if not impossible, to discern whether N_2_ fixation rate estimates in previous studies may have been confounded due to the assimilation of ^15^N contaminants in ^15^N_2_ gas stocks. Given that ^15^N_2_ stocks from only one of the three suppliers tested here contained contaminants to an extent that would interfere with any but the lowest reported N_2_ fixation measurements, there is a strong likelihood that published estimates performed with ^15^N_2_ from the other two suppliers have not been significantly inflated by labeled contaminants. In fact, many estimates may be lower than reality due to the incomplete equilibration of ^15^N_2_ gas with the incubation medium, a pervasive problem with aqueous ^15^N_2_ fixation assays that was diagnosed only recently [41]–[43]. Nevertheless, it is advisable at this point to analyze commercial ^15^N_2_ stocks prior to their use to ensure their relative purity. In doing so, particular attention must be paid to the lower limit of detection for N_2_ fixation rates. In recent years, workers have reported estimates of very low rates (≤0.1 nmol L^−1 ^d^−1^) in environments where N_2_ fixation is otherwise unexpected, which include oxygen-deplete regions of the water column at Pacific margins [34,35,38], as well as in the Beaufort Gyre of the Arctic Ocean [36]. Such minimal rates are questionable, considering that the relatively clean Cambridge Isotopes ^15^N_2_ gas was found to contain enough ^15^N-ammonium to infer N_2_ fixation rates of up to 0.02 nmoles N L^−1 ^d^−1^. Campro Scientific and other commercially available ^15^N_2_ gas stocks could similarly contain minute, but significant, concentrations of ^15^N-nitrate or ammonium. Therefore, it behooves investigators to not only verify the purity of their commercial ^15^N_2_ prior to use, but also to generate constraints as to the lower limit of detection, allowing for the possibility that a trace-level ^15^N-contaminant could interfere with the detection of diminutive N_2_ fixation rates.

### Steps toward mitigation

The catalytic synthesis of ^15^N_2_ gas from ^15^N-ammonia gas invariably entails the incidence of ^15^N-ammonium and ^15^N^–^NOx contaminants, the removal of which is dependent on the stringency of scrubbing procedures to which a given batch is subjected. The consistency of ^15^N-nitrate/nitrite measurements among bottles from an individual lot from Sigma-Aldrich (*Sigma A1-A3*), in contrast to the lower ^15^N-nitrate/nitrite detected in a subsequent lot (*Sigma B*), supports the premise that the levels of ^15^N-contaminants are associated with discrete batch syntheses, identified by lot numbers, rather than with individual lecture bottles. The variability in ^15^N-ammonium among lecture bottles of the same lot suggests that ammonia gas does not disperse homogeneously in compressed N_2_ gas. In any case, large-scale batch syntheses of ^15^N_2_ occur relatively infrequently, on the order of every 2 years at Isotec (subsidiary of Sigma-Aldrich). We currently have a verbal agreement with Isotec to perform nitrate and ammonium isotopic analyses of ^15^N_2_ batches, toward providing a certificate of analysis ensuring adequate purity for N_2_ fixation assays. In the meantime, we advise that workers procure low-contaminant stocks from lots that we tested here. The very high purity of the batches from these suppliers suggests stringent and efficacious purification protocols, such that batches synthesized by these groups in the future are likely to be equally pure, notwithstanding the potential for human error during synthesis or subsequent purification.

Regardless of ‘expected’ purity, we recommend that workers explicitly test new batches availed by respective suppliers for ^15^N-nitrate and ammonium prior to using them in N_2_ fixation assays, and actively disseminate the results to targeted web-based forums. To test a given batch, ^15^N_2_ gas can be equilibrated with nitrate and ammonium solutions following protocols akin to the low and high sensitivity equilibrations herein. A number of laboratories perform commercial nitrate isotope analyses routinely at a modest cost per sample. Ammonium isotope analyses are substantially more involved, but are also performed routinely by a number of laboratories.

We further recommend that pertinent publications include not only the brand of ^15^N_2_ stock, but also the associated lot number, and references to reported contaminants. With continued testing, our understanding of the prevalence of commercial ^15^N_2_ contamination will grow and shed light on this problem, which may have plagued N_2_ fixation estimates in the past.
